# Supplementary material for: Predicting organoid morphology through a phase field model: Insights into cell division and lumenal pressure
Source: PLoS Comput Biol. 2025 Aug 18;21(8):e1012090. doi: 10.1371/journal.pcbi.1012090 (PMC12373292; doi:10.1371/journal.pcbi.1012090)
Supplement: S1 Text — This file contains supplementary analyses presented in the following sections: (1) Parameters in cell dynamics – the influence of parameter variations on cell shape and growth dynamics in the phase-field model; (2) Morphology sensitivity to phase-field parameters – the sensitivity of morphological outcomes to changes in phase-field parameters; (3) Initial cell numbers and morphology – the impact of initial cell numbers on organoid morphology. The following figures are associated with these sections: (Fig A) Cell shapes under parameter variation – analysis of cell shape under different values of α, β, γ, and η. (Fig B) Phase diagrams with slightly varied parameters – the phase diagrams remain consistent in appearance across different settings of α, β, γ, and η. (Fig C–G) Parameter sensitivity of each morphology—indices are plotted across varying values of α, β, γ, and η for different morphologies. (Fig H–I) Phase diagram of organoid morphology for η=0.002–0.010. (Fig J–N) Indices as ξ, td, and η vary. (Fig O) Morphologies resulting from different initial cell numbers — simulations starting from two and seven initial cells are compared with the four-cell condition used in the main text. These results provide additional insights that complement the findings in the main text. (PDF) [file pcbi.1012090.s001.pdf]

S1 Text: Supporting Information
Predicting organoid morphology through a phase field model: Insights into cell division and lumenal pressure

This document describes supplementary results that complement the findings presented in the main manuscript.

1 Parameters in cell dynamics

To investigate the impact of parameter variations on cell shape and growth within our phase-field model, we conducted simulations with diverse parameter settings. In all cases, the initial configuration comprised seven cells, each at half of their target volume, $V_{\text{target}} = 3$. The setup included one cell at the center with coordinates $(0, 0)$, labeled as $n = 0$, and six others symmetrically arranged at $(0.5\cos(n\theta), 0.5\sin(n\theta))$, where $n = 1, 2, \ldots, 6$, and $\theta = \frac{2\pi}{6}$. Assuming circular shapes for cells, we assigned $u_n = 1$ within a radius of $\sqrt{\frac{V_{\text{target}}}{2\pi}} \sim 0.69$ from each cell's center. This design facilitated the study of the relaxation process and steady-state conditions arising from initially overlapping cells, with a specific focus on the impact of parameters rather than cell division dynamics. We varied one parameter at a time - α, β, γ, or η - while keeping the others constant at $\alpha = 1$, $\beta = 1$, $\gamma = 0.1$, and $\eta = 0.08$.

Figs A(a-c) illustrates the results under varying α, a parameter connected to the rate of cell volume growth [see Modeling approach section in the main text]. At steady state, the cell shapes showed minimal variation across different α values, evident in the organoid cross-sections on the y-axis [Fig A(a)]. However, the rate of volume increase and the steady-state volume of the central cell displayed notable differences with varying α. Larger α values led to a more rapid increase in cell volume, with the time to reach $V_d = 2.9$ decreasing correspondingly [Fig A(c)]. The cell at $\alpha = 0.4$ did not exceed the volume of V_d, but a general upward trend in steady-state volume was observed with increasing α [Fig A(b)].

Figs A(d-f) showcases the results from simulations with varying values of β, a parameter that influences the volume exclusion effect [refer toModeling approach section in the main text]. Changes in β predominantly affected the distances between cells, with an increase in β resulting in greater separation. Despite these variations in distance, the overall shapes of the cells remained consistent across different values of β [Fig A(d)]. The steady-state volume of the central cell showed little variation across different β levels [Fig A(e)]. With $\beta \leq 0.4$, the pattern of volume increment exhibited complexity; generally, the volume increased over time but experienced a slight decrease before reaching saturation. Moreover, at $\beta > 1$, the volume initially decreases before increasing. Consequently, the time required for the volume to reach $V_d = 2.9$ demonstrated a concave relationship relative to increasing β, as illustrated in Fig A(f).

In Figs A(g-i), we explored the effects of varying γ, a parameter influencing surface tension. Note that γ must exceed η for accurate modeling [refer to Modeling approach section in the main text]. The results showed that as γ increased, the distance between cells expanded, and the area of overlap between cells decreased [Fig A(g)]. With higher γ values, The central cell's steady-state volume tended to decrease [Fig A(h)]. In addition, while the initial phase of growth was faster, the slowdown near the saturation point was more pronounced, as larger γ values. This led to an increase in the time required for the cell volume to reach $V_d = 2.9$, as illustrated in Fig A(i).

Lastly, Figs A(j-l) demonstrates the outcomes from simulations with different η values, a parameter associated with cell-cell adhesion. Here, it is important that γ remains greater than η.

Fig A. Cell shapes and growth with varying parameters. Each column in the figure displays, from left to right, the cross-sections of an organoid at the y-axis, the volume of the central cell $V_c(t)$ with the inset of $V_{target} - V_c(t)$ where $V_{target} = 3$, and the time taken for the volume to exceed V_d, respectively. Each row corresponds to the results obtained by varying the parameters: α (a-c), β (d-f), γ (g-i), and η (j-l).

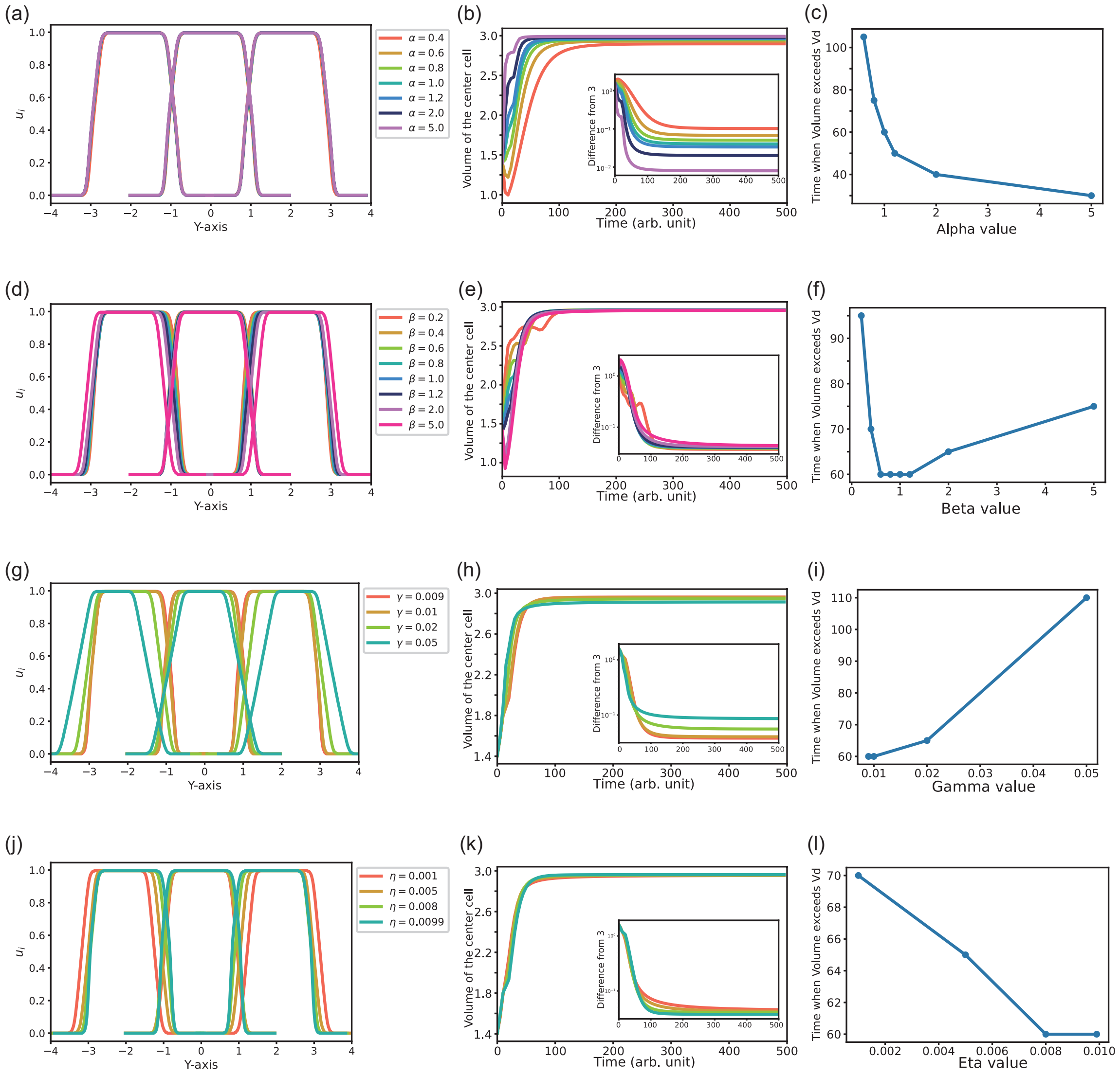

Unlike the trends observed with increasing γ, increasing η led to opposite effects: the distance between cells decreased, and the overlap area increased as η was raised [Fig A(j)]. The central cell's steady-state volume showed an increase, and its initial growth rate slowed down as η increased [Fig A(k)]. This slowdown at the point close to saturation became less pronounced with larger η values, thereby decreasing the time for the volume to reach $V_d = 2.9$ [Fig A(l)].

2 Morphology sensitivity for the phase field parameters

In this model, nine parameters are involved, including D, η, γ, α, β, ξ, t_d, V_d and τ. Through non-dimensionalization, three reference scales (L, P, and v in the main text) were introduced, reducing the number of independent parameters to six. Among these, in the main text, we constructed phase diagrams by systematically varying ξ and t_d, treating them as the primary variables. Consequently, the remaining four parameters need to be treated as independent. While there is some arbitrariness in choosing which parameters to treat as independent, we chose to focus on α, β, γ, and η—parameters specific to the phase field formulation introduced for simulating organoid growth.

Those used in simulations must be chosen carefully to suit the study purpose. In addition, we need to know how robust the phase diagram is. In this section, we conduct a sensitivity analysis to evaluate the validity of the parameter values used in the main text.

2.1 One-at-a-time (OAT) approach

There are various methods for sensitivity analysis; first, we adopt the one-at-a-time (OAT) approach. In this method, three of the four parameters are fixed at their central values, and one parameter is varied at a time. For each case, we compute morphology-related output metrics to identify the parameter ranges in which characteristic morphologies appear robustly.

Detailed results are presented in the following subsections. In summary, the parameter choices used in the main text are sufficiently robust. Morphologies are particularly sensitive to changes in α at a short t_d, but even then, the qualitative structure of the phase diagram—specifically, the relative positioning of the morphological phases—remains unchanged. Therefore, we conclude that ξ and t_d are the primary determinants of morphogenesis, and it is reasonable to construct the phase diagram by varying these two parameters.

Overview of the sensitivity for all morphologies

First, we tested the sensitivity of the phase diagram to small changes in the parameters (Fig B(a-f)). Second, we took the OAT approach by fixing three parameters at their central values and varying one parameter in wider ranges. (Fig C–G)

The results show that the boundaries between any two phases shift, but the overall appearance of the phase diagram remains unchanged (Fig B). Therefore, we can conclude that the phase diagram is robust with respect to these parameters.

Then, we investigated the general influence of the parameter α on organoid growth and morphology. α represents the strength of cell growth. When $\alpha \leq 0.4$, cells do not grow in any configuration and remain in their initial state, resulting in a failure to develop organoids. In the range of 0.5 to 0.7, the cell sheet tends to rupture during the early stages of development. As α increases to values around 0.7, distinct morphological patterns emerge across almost all configurations. Around $0.8 < \alpha < 1.2$, most morphologies maintain their structural pattern. Above around $\alpha = 1.2$, multilayer no-stable-lumen tends to emerge in morphologies with $t_d = 40$. For $t_d = 120$, each pattern gradually shifts to a multilayer no-stable-lumen. For $\alpha > 5$, the cell growth rate becomes extremely rapid, causing cells to invade neighboring regions beyond the constraints imposed by the excluded volume effect. However, an interesting exception is that monolayer cysts do not shift to multilayered morphology even if α is so large. In summary, although varying α changes the magnitude of cellular pressure and thus the relative pressure exerted by the lumen, the qualitative structure of the phase diagram remains unchanged [Figs B(a-c) and Fig C–G].

Next, we examined the general influence of the parameter β, which governs the excluded volume effect. When $\beta = 0.1$, the volume exclusion is so weak that cells and lumens overlap, preventing the formation of an organized structure. At $\beta = 0.2$, the boundaries between cells and the lumen are still partially overlapping; however, organoid growth proceeds. As β increases, the boundary between cells and the lumen becomes more distinct, though the overall organoid can grow.

Fig B. Phase diagrams with slightly varied parameters. The phase diagrams remain consistent in appearance across different parameter settings: (a) $\alpha = 0.9, \beta = 1.0, \gamma = 0.01, \eta = 0.008$; (b) $\alpha = 1.0, \beta = 1.0, \gamma = 0.01, \eta = 0.008$ (same as in the main text); (c) $\alpha = 1.1, \beta = 1.0, \gamma = 0.01, \eta = 0.008$; (d) $\alpha = 1.0, \beta = 0.6, \gamma = 0.01, \eta = 0.008$; (e) $\alpha = 1.0, \beta = 1.0, \gamma = 0.01, \eta = 0.006$; (f) $\alpha = 1.0, \beta = 1.0, \gamma = 0.008, \eta = 0.006$. The simulation boundary was a square with a size of 20×20, which corresponds to half the length of Fig 3 in the main text. Due to randomness, the phases in (b) exhibit slight differences, even though the same parameter set as in Fig 3 of the main text is used. Nevertheless, the overall shape of the phase diagram was not affected by these small parameter variations. For certain parameter sets where the lumen has leaked but the previous shape is preserved, the final morphology is shown.

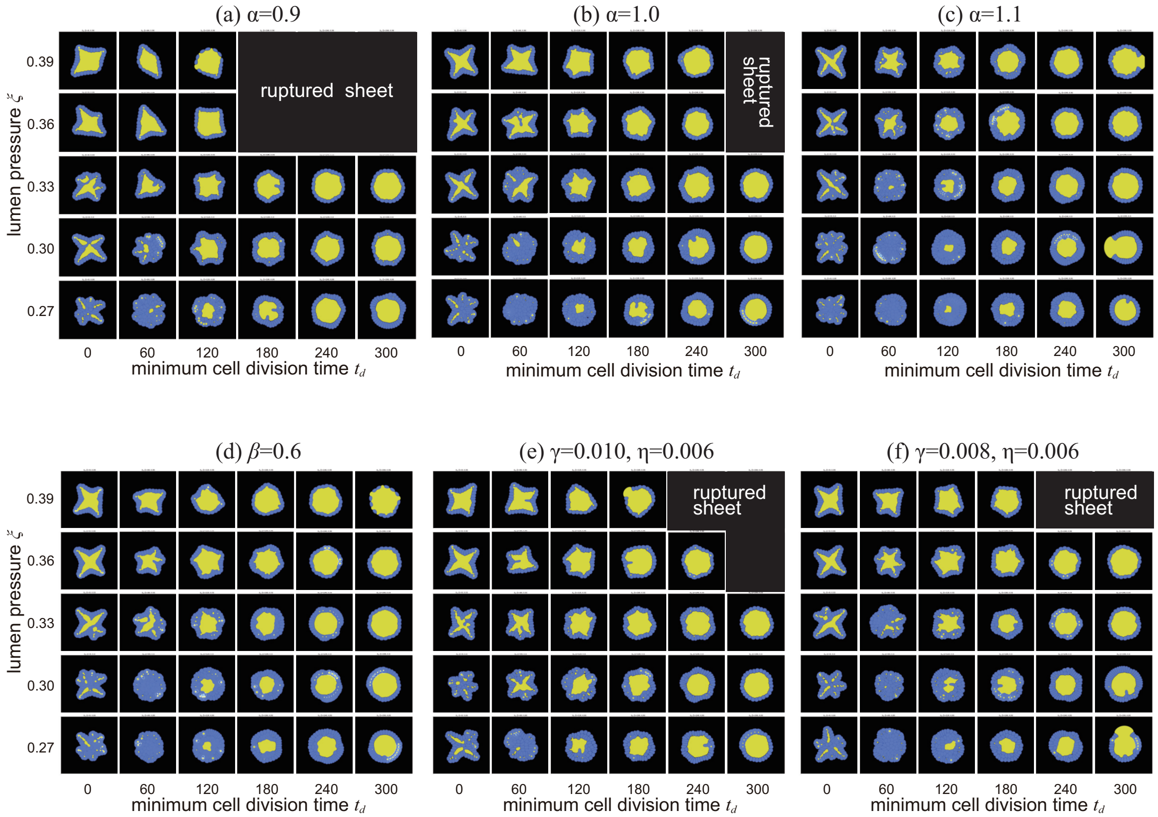

When γ is large, the lumen tends to become larger. This is because a high γ increases the surface tension of the phase field variable, promoting rounder cell shapes and thereby effectively weakening cell–cell adhesion. As a result, similar morphological patterns emerge as in the case of small η.

Star-shaped structure

We investigated the star-shaped morphology under the condition of $\xi = 0.37$ and $t_d = 40$ with varying $\alpha, \beta, \gamma, \eta$, and found that this configuration is highly sensitive to variations in system parameters.

Fig C. Parameter sensitivity of star-shaped morphology. Regions without shading indicate parameter regimes where the star shape morphology is observed. Shaded areas indicate regimes in which other morphologies emerge.

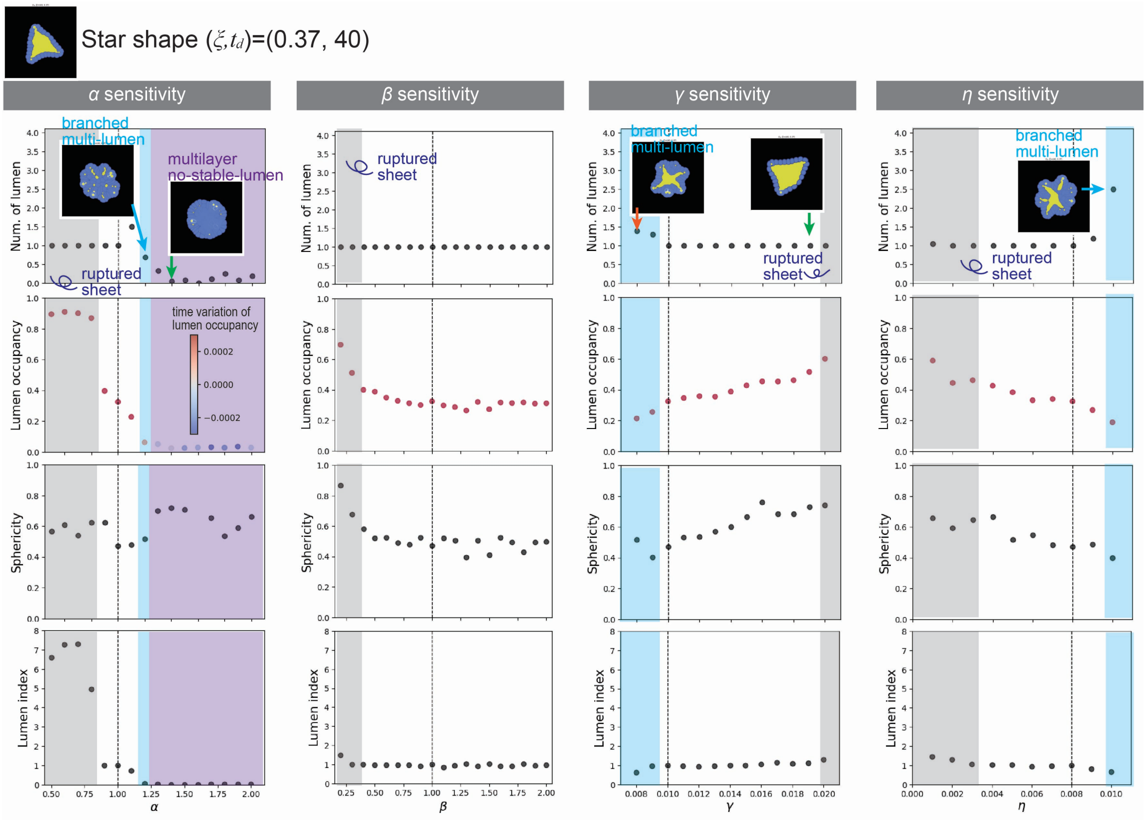

When α is in the range of 0.5 to 0.8, the cell sheet tends to rupture, because the pressure from cellular growth is insufficient to counteract lumen expansion (gray region in Fig C). The star-shaped morphology is maintained within the range $0.9 \leq \alpha \leq 1.1$. At $\alpha = 1.2$, the cell layer becomes significantly thicker, leading to the emergence of a branched multi-lumen structure (blue region in Fig C). For $\alpha > 1.2$, the lumen disappears and the system transitions into a multilayer no-stable-lumen morphology, suggesting that the cellular pressure overcomes lumen growth (purple region in Fig C).

With respect to β, values of $\beta \leq 0.2$ cause the cell sheet to rupture due to insufficient excluded volume effect (gray region in Fig C). In the other region, the star-shaped morphology is maintained.

For the parameter γ, when $\gamma \leq 0.009$, the system tends to develop thicker cell layers, which in turn promote the formation of branched multi-lumen structures (blue region in Fig C). However, when $\gamma > 0.02$, the cell sheet becomes unstable and breaks (gray region in Fig C).

Regarding cell-cell adhesion, quantified by η, values of $\eta \leq 0.003$ are insufficient to maintain sheet integrity, resulting in a ruptured sheet (gray region in Fig C). On the other hand, for $\eta \geq 0.1$, strong adhesion suppresses lumen expansion, thus promoting the formation of branched multi-lumen morphologies (blue region in Fig C).

As discussed in the main text, sustaining a single-layered cell sheet typically requires a lumen

index of 1. Achieving and maintaining such configurations without specialized feedback mechanisms remains difficult.

Monolayer cyst structure

We investigated the robustness of the monolayer cyst morphology under the condition of $\xi = 0.33$ and $t_d = 280$ with varying $\alpha, \beta, \gamma, \eta$, and found it to be relatively insensitive to variations in mechanical parameters. For the volume elasticity α, when $\alpha \leq 0.8$, the cell sheet tends to rupture,

Fig D. Parameter sensitivity of monolayer cyst morphology. Regions without shading indicate parameter regimes where the monolayer cyst morphology is observed. Shaded areas indicate regimes in which other morphologies emerge. The wavy line on the x-axis indicates a break in the scale

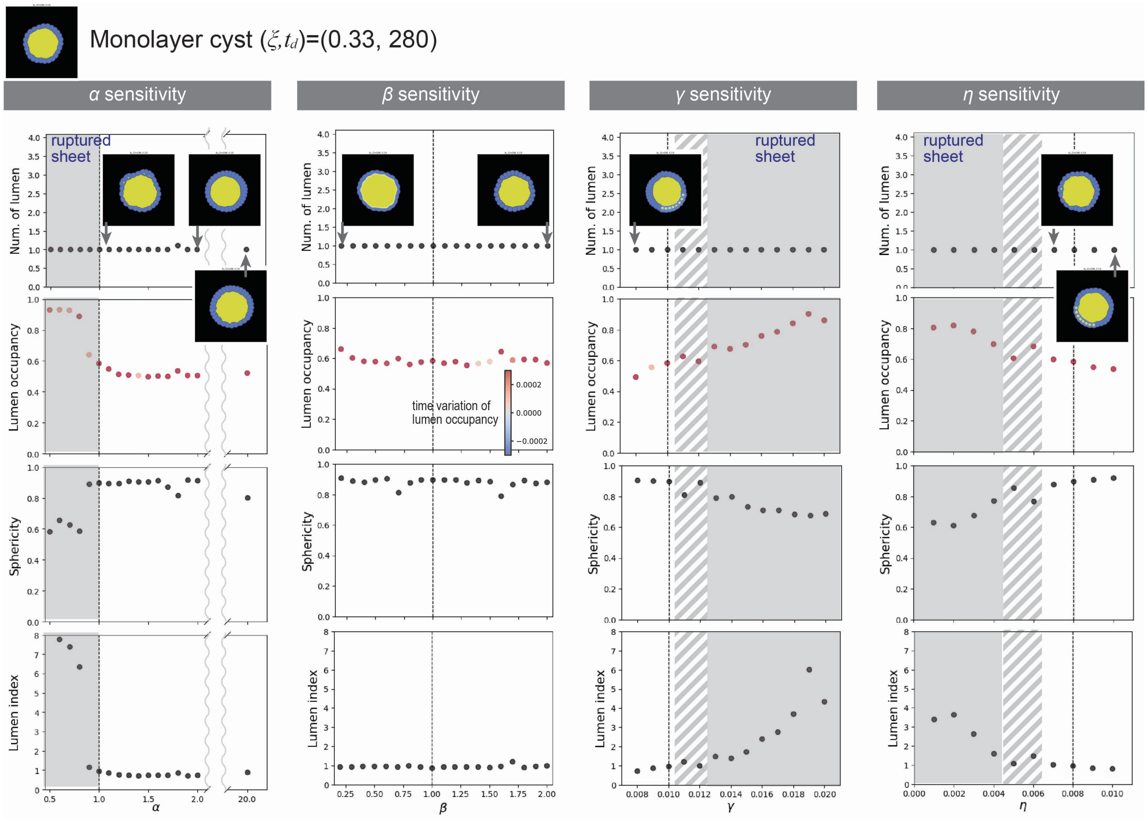

as observed in other morphological types (gray region in Fig D). However, for $\alpha \geq 0.9$, the monolayer cyst structure remains intact, even when α is increased substantially. We confirmed that the morphology remains stable even at $\alpha = 20$ (data not shown), indicating high robustness to variations in this parameter.

For the volume exclusion parameter β, monolayer cysts stably emerge for values $\beta \geq 0.2$, which lies within a biologically meaningful range.

Regarding the cortical tension γ, monolayer cysts form stably for values between $\gamma = 0.008$ and $\gamma = 0.01$. When γ is increased slightly to 0.011 or 0.012, the system shows probabilistic outcomes—sometimes maintaining the cyst structure, other times resulting in rupture (gray striped region in Fig D). At $\gamma \geq 0.013$, rupture is observed consistently (gray region in Fig D). This behavior is attributed to increased surface tension, which effectively weakens cell–cell adhesion.

A similar trend is observed for the cell–cell adhesion parameter η, but in the opposite direction. For $\eta \leq 0.004$, the monolayer structure ruptures (gray region in Fig D). At $\eta = 0.005$ or 0.006,

rupture occurs probabilistically (gray striped region in Fig D), whereas for $\eta > 0.006$, the monolayer cyst remains stable.

Branched multi-lumen

We analyzed the parameter sensitivity of the branched multi-lumen morphology under the condition of $\xi = 0.32$ and $t_d = 40$ with varying $\alpha, \beta, \gamma, \eta$. This morphology exhibits relatively high sensitivity, which is consistent with the frequent appearance of this pattern in other parameter regimes.

Fig E. Parameter sensitivity of branched multi-lumen morphology. Regions without shading indicate parameter regimes where the branched multi-lumen morphology is observed. Shaded areas indicate regimes in which other morphologies emerge.

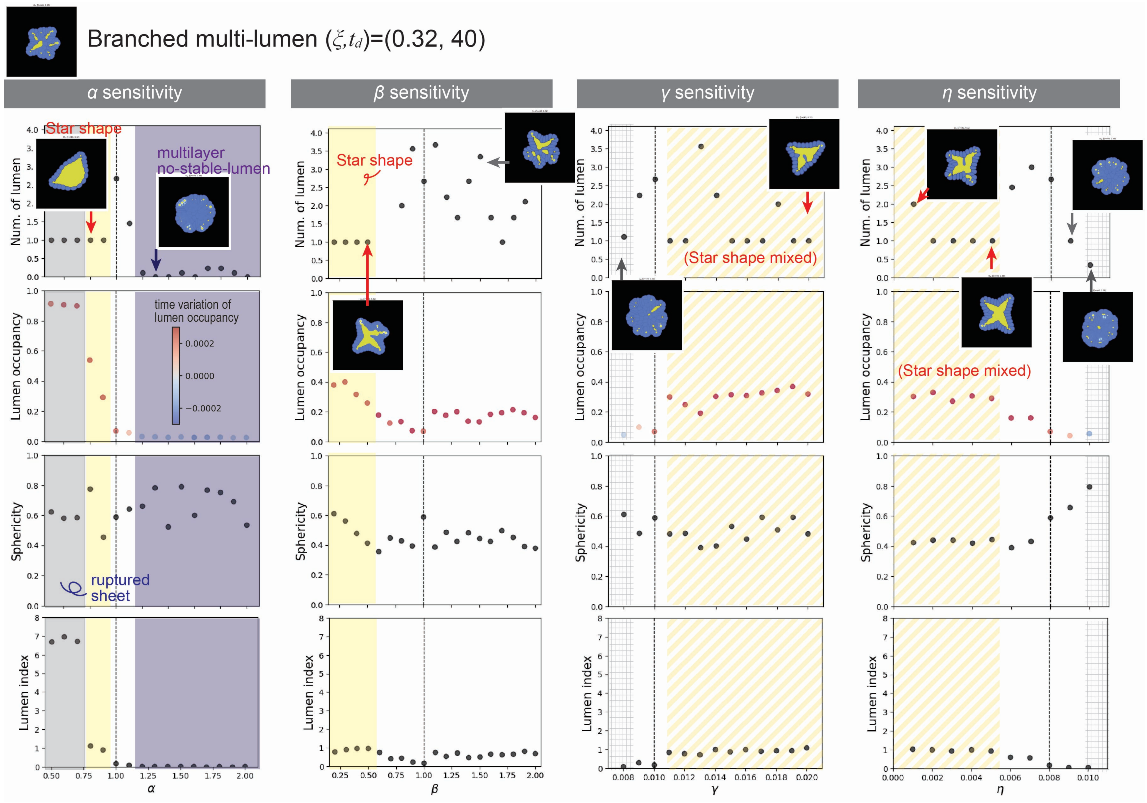

When α is in the range of 0.5 to 0.7, the cell sheet ruptures (gray region in Fig E). At $\alpha = 0.8$ and 0.9, a star-shaped morphology emerges (yellow region in Fig E). It is because the lumen pressure is stronger than the cell growth pressure, and lumen can grow faster than branched multilumen. For $\alpha = 1.0$ and 1.1, the branched multi-lumen morphology is preserved. When $\alpha \geq 1.2$, the morphology transitions into a multi-layer no-stable lumen structure, which is the same as the star shape (purple region in Fig E).

For the excluded volume parameter β, values below 0.5 result in a transition to the star-shaped morphology. It is because weak volume exclusion allows the lumen get in between the interval between cells.

At $\gamma = 0.008$, a multi-layer single-stable lumen structure appears, although its final morphology closely resembles a branched multi-lumen (cross-hatched region in the diagram in Fig E). When $\gamma \geq 0.011$, either a star-shaped or a branched multi-lumen morphology appears probabilistically (the yellow striped region in Fig E).

Regarding cell-cell adhesion η, values below 0.005 result in the probabilistic appearance of the star-shaped morphology (yellow striped region in Fig E). In contrast, when $\eta \geq 0.01$, the morphology

is classified as a multi-layer single-stable lumen, but observation of the final state suggests that multiple lumens appear transiently and eventually diminish over time (cross-hatched region in the diagram in Fig E).

Multilayer multi-lumen

The multilayer multi-lumen morphology does not emerge unless the system size is sufficiently large. In the early stages of development, when the organoid is still small, it typically contains either zero or one lumen. However, as the organoid grows larger, it can acquire the capacity to support multiple lumens. Indeed, we confirmed that the multilayer multi-lumen morphology does not appear when the organoid size is insufficient, even when the parameters are set to the corresponding case of Fig 3 in the main text. In this way, the observed morphology is sensitive to organoid size, i.e., when to observe the morphology, especially near the parameter range where the multilayered multi-lumen structure emerges in a large organoid.

Multilayer no-stable-lumen

We examined the parameter dependence of the multi-layer no-stablelumen morphology under the condition of $\xi = 0.27$ and $t_d = 60$ with varying $\alpha, \beta, \gamma, \eta$. Our observations indicate that this morphology tends to shift toward a branched multi-lumen configuration depending on the values of specific parameters.

Fig F. Parameter sensitivity of multilayer no-stable-lumen morphology. Regions without shading indicate parameter regimes where the multilayer no-stable-lumen morphology is observed. Shaded areas indicate regimes in which other morphologies emerge.

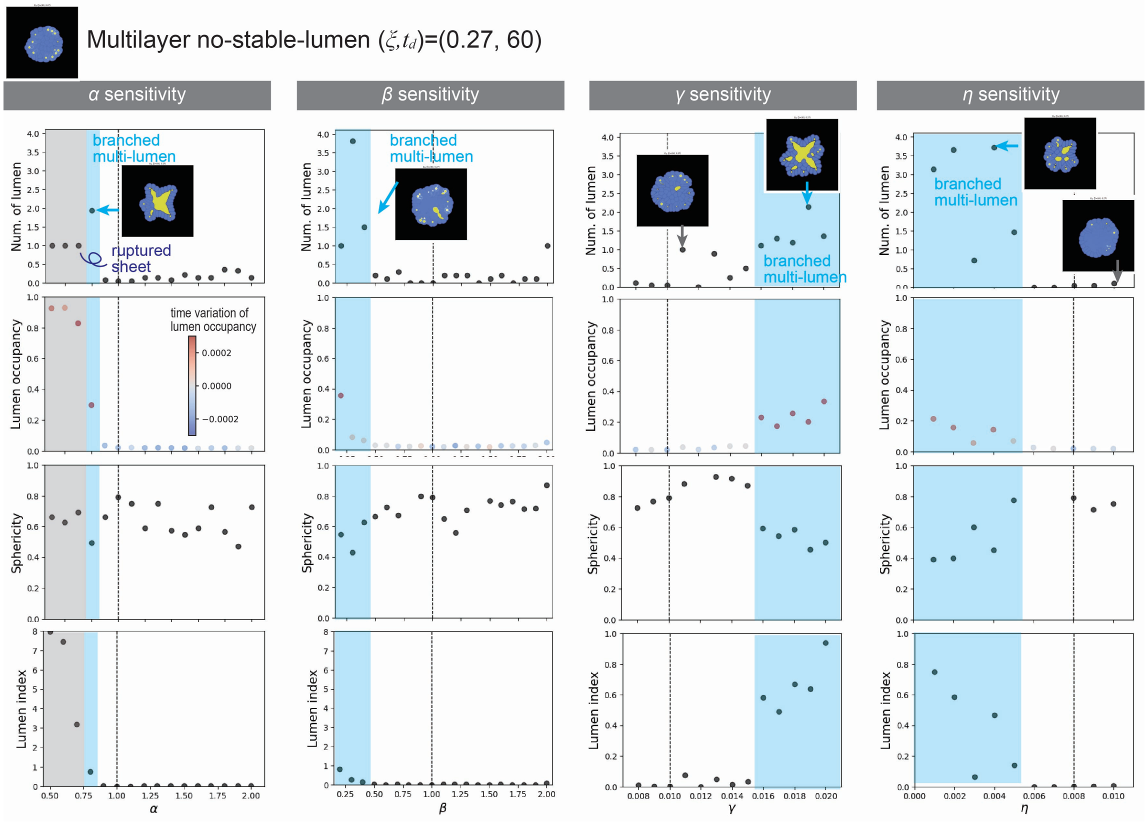

When α is in the range of 0.5 to 0.7, the cell sheet tends to rupture, as many other morphologies do (gray region in Fig F). At $\alpha = 0.8$, a branched multi-lumen structure appears, which can be attributed to the dominance of the internal pressure of the lumen over the outward pressure generated by cell growth, consistent with behaviors observed in other morphologies (blue region in Fig F).

Regarding the excluded volume parameter β, when $\beta \leq 0.4$, branched multi-lumen structures emerge (blue region in Fig F). This behavior is due to weakened excluded volume effects, which allow lumens to infiltrate the spaces between cells more easily.

As for γ, values equal to or greater than 0.016 promote the formation of branched multi-lumen morphologies (blue region in Fig F).

In the case of the adhesion parameter η, values below 0.005 also lead to branched multi-lumen structures (blue region in Fig F). This is likely because weak intercellular adhesion facilitates the expansion of lumens.

Multi-layer single-stable lumen structure

Under the condition of $\xi = 0.27$ and $t_d = 120$, the multilayer single-stable-lumen structure appears as a relatively stable morphology within the standard parameter range of α, β, γ, and η.

Fig G. Parameter sensitivity of multilayer single-stable lumen morphology.

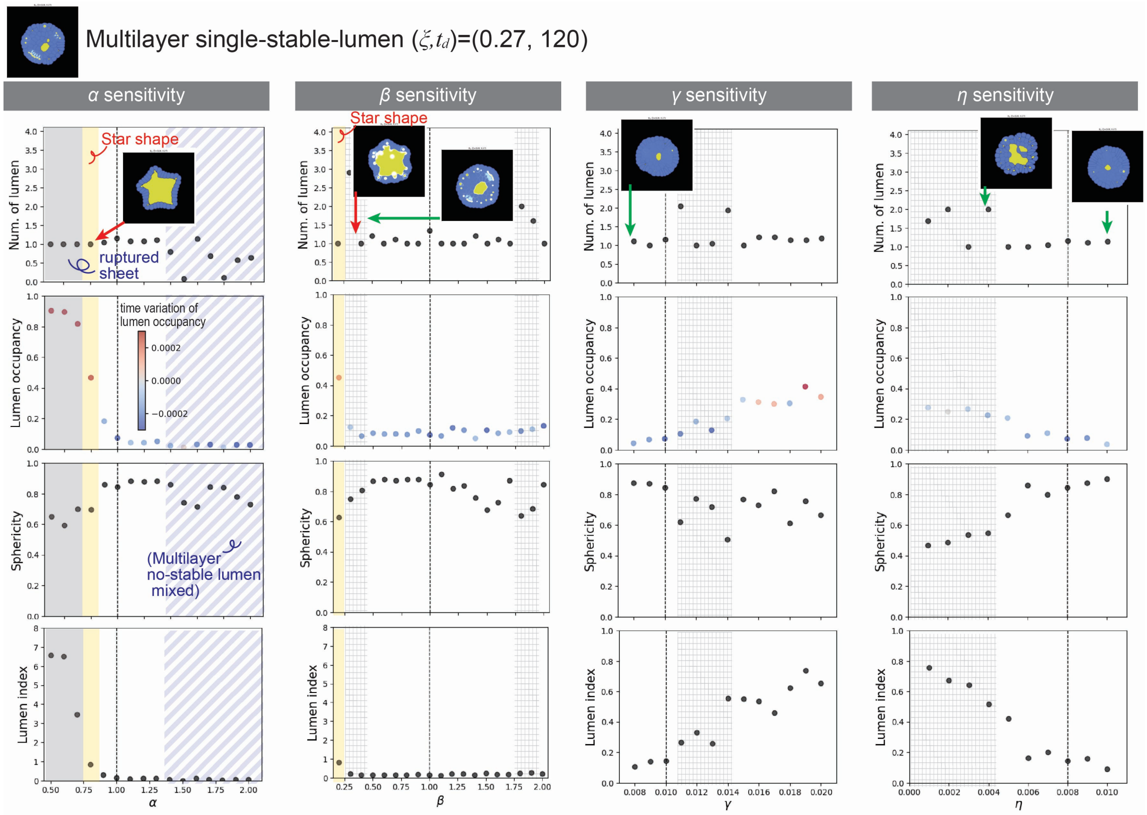

When α is in the range of 0.5 to 0.7, the cell sheet tends to rupture. At $\alpha = 0.8$, a star-shaped morphology emerges, which can be attributed to the lumen pressure exceeding the pressure exerted by the growing cells. Around $\alpha = 1$, the multilayer single-stable lumen morphology persists. For $\alpha > 1.5$, the multilayer non-stable lumen structure stochastically forms (purple stripe region in Fig G).

When $\beta = 0.2$, star-shaped structures also appear (yellow region in Fig G). A smaller β implies a weaker excluded volume effect, allowing the lumen to infiltrate the spaces between cells more easily and expand more readily. At some β values like 0.3, 0.4, 1.8, and 1.9, the multilayer multilumen pattern stochastically appears judging from indicies, but it will go to the multilayer single-stable-lumen morphology (cross-hatched region region in Fig G). Other values of β do not significantly alter the morphology.

The morphology remains generally stable against changes in γ and η. Although multiple lumens may transiently emerge due to randomness, it is expected that they eventually merge into a single lumen over time (cross-hatched region region in Fig G).

2.2 Multiple-parameter approach

Next, we performed a multiple-parameter sensitivity analysis by running a total of 900 simulations, systematically varying three parameters: the minimum cell division time (t_d), the luminal pressure (ξ), and the cell–cell adhesion (η). Specifically, t_d was varied from 30 to 300 in steps of 30, ξ from 0.22 to 0.4 in steps of 0.02, and η from 0.002 to 0.010 in steps of 0.001. All simulations were conducted using a reduced system whose edge length was half that of the main system described in the main text (i.e., an overall volume four times smaller).

Figs H and Figs I correspond to the main text's Fig 3, showing phase diagrams for various values of η. Overall, we observed that decreasing η shifts the entire phase diagram downward, with this effect being particularly pronounced for the region corresponding to the ruptured sheet state. Compared to the single-layer region, the multilayer region does not shift downward as much. Snapshots also show that individual cells become rounder as η decreases.

These observations are likely due to weaker cell–cell adhesion at lower η. In addition, η influences the distribution of the cortical force generator that determines the division plane angle. At the original value of $\eta = 0.008$, cells tend to divide along the tangential direction within the layer. As η decreases, this orientation becomes more random, which increases the likelihood of multilayer formation.

Fig H. Phase diagram of the organoid morphology at $\eta = 0.002 - 0.005$. Each color domain corresponds to: ruptured sheet (black) star shape (yellow), monolayer cyst (green), branched multi-lumen (blue), multilayer multi-lumen (red), multilayer no-stable-lumen (purple), and multilayer single-stable-lumen (gray).

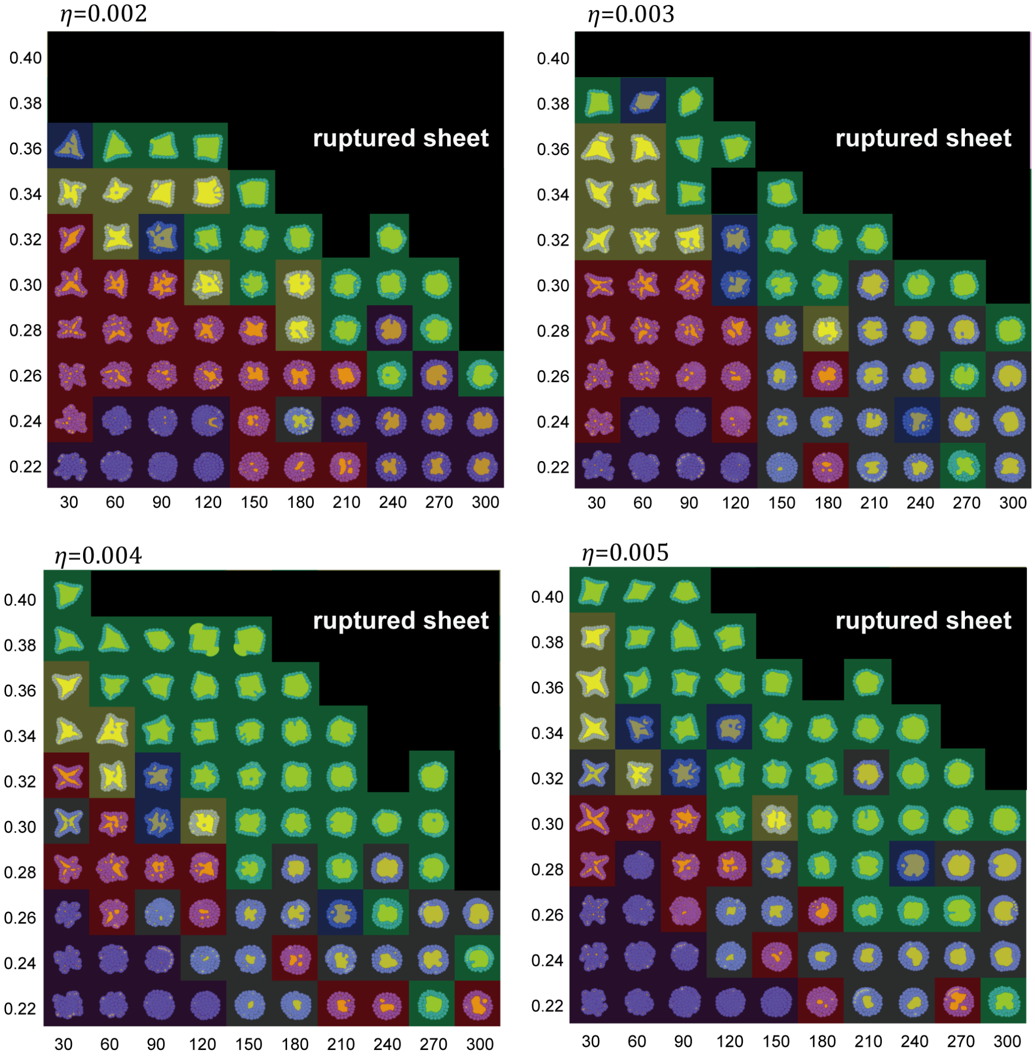

Fig I. Phase diagram of the organoid morphology at $\eta = 0.006 - 0.010$. Each color domain corresponds to: ruptured sheet (black) star shape (yellow), monolayer cyst (green), branched multi-lumen (blue), multilayer multi-lumen (red), multilayer no-stable-lumen (purple), and multilayer single-stable-lumen (gray).

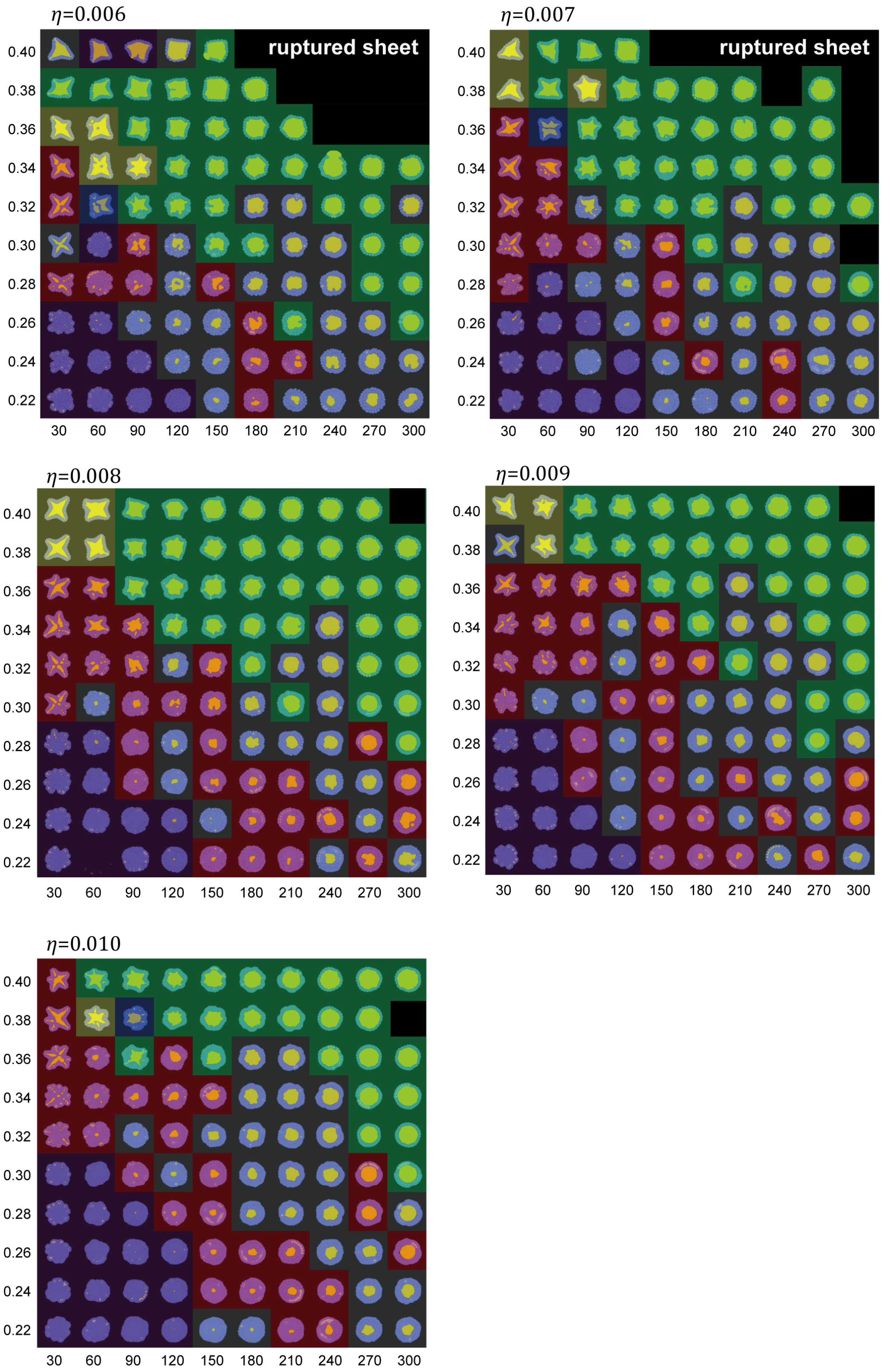

Figs J–N simultaneously visualize five quantities for all 900 parameter combinations: sphericity, number of lumens, lumen occupancy, change in lumen occupancy, and lumen index difference. In each plot, the vertical axis represents ξ and the horizontal axis represents the observation time t_d. At each intersection of ξ and t_d, a mini-grid of nine squares is shown, corresponding to the nine values of η tested under the same ξ–t_d condition. Within each mini-grid, the arrangement from top left to top right and then row by row is $\eta = 0.002$, 0.003, 0.004, then the next row starts with 0.005, and so on.

Fig J. Sphericity for various parameter sets. Each mini-grid shows sphericity for nine η values at each ξ–t_d coordinate; color represents the sphericity level. Black squares indicate the ruptured sheet state, while white squares correspond to cases where no lumen was formed and sphericity could not be computed. The nine η values within each mini-grid are arranged as follows: the top row shows $\eta = 0.002$, 0.003, and 0.004 from left to right; the middle row shows 0.005, 0.006, and 0.007; and the bottom row shows 0.008, 0.009, and 0.010.

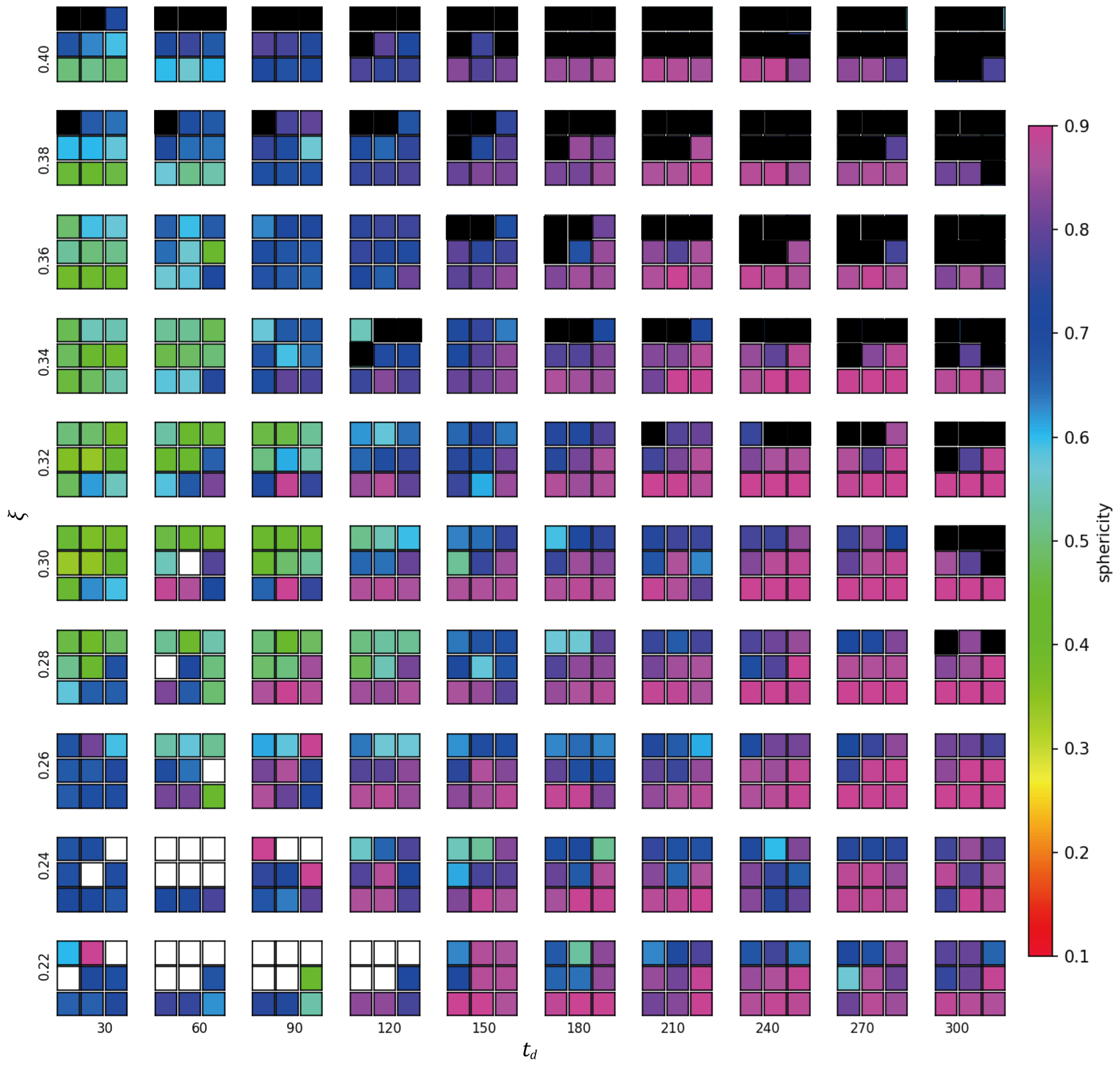

Fig K. Number of lumens for various parameter sets. Each mini-grid shows the number of lumens for nine η values at each ξ–t_d coordinate; color represents the lumen count. Black squares indicate the ruptured sheet state. The nine η values within each mini-grid are arranged as follows: the top row shows $\eta = 0.002$, 0.003, and 0.004 from left to right; the middle row shows 0.005, 0.006, and 0.007; and the bottom row shows 0.008, 0.009, and 0.010.

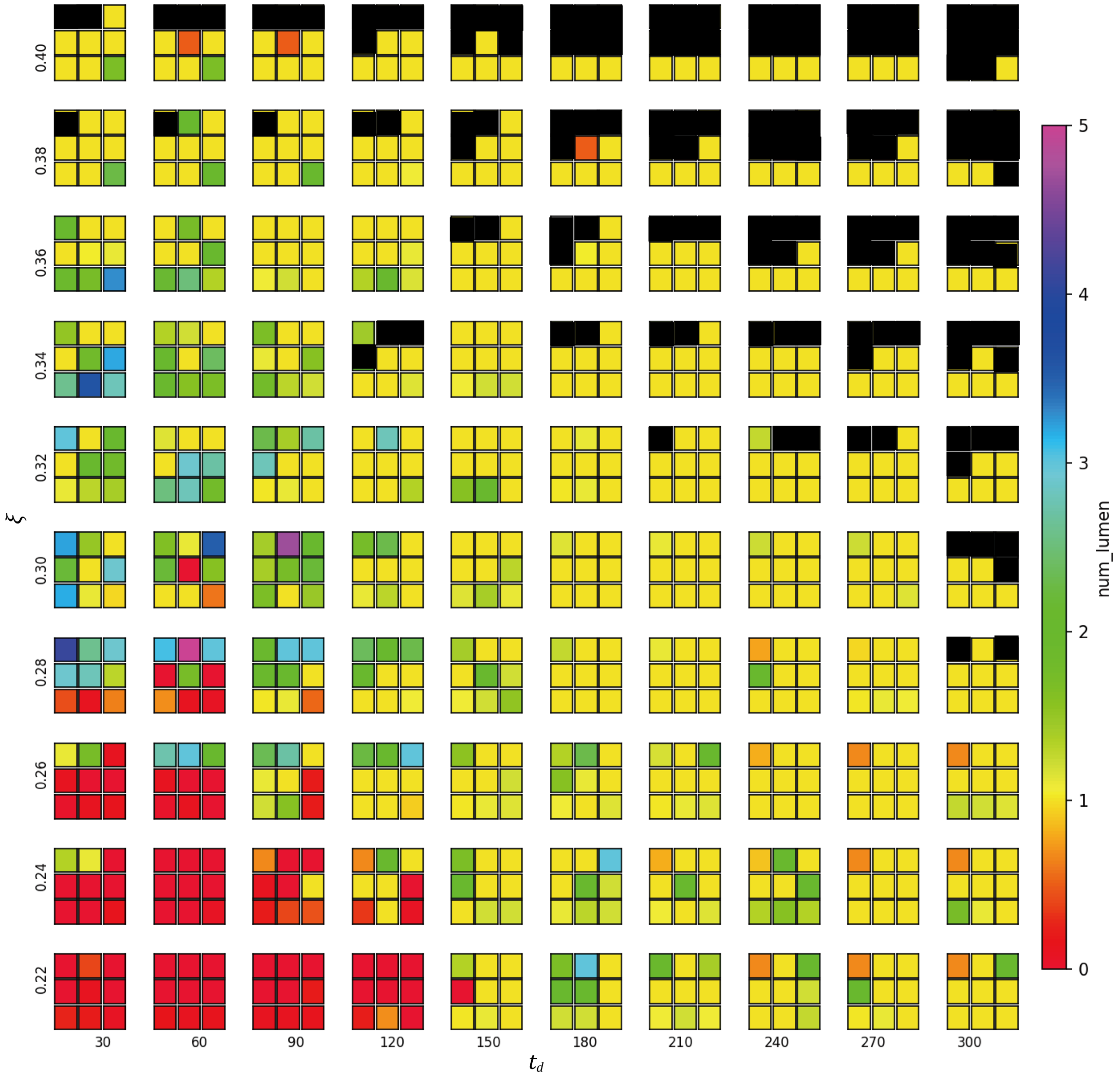

Fig L. Lumen occupancy for various parameter sets. Each mini-grid shows lumen occupancy for nine η values at each ξ–t_d coordinate; color represents the occupancy level. Black squares indicate the ruptured sheet state. The nine η values within each mini-grid are arranged as follows: the top row shows $\eta = 0.002$, 0.003, and 0.004 from left to right; the middle row shows 0.005, 0.006, and 0.007; and the bottom row shows 0.008, 0.009, and 0.010.

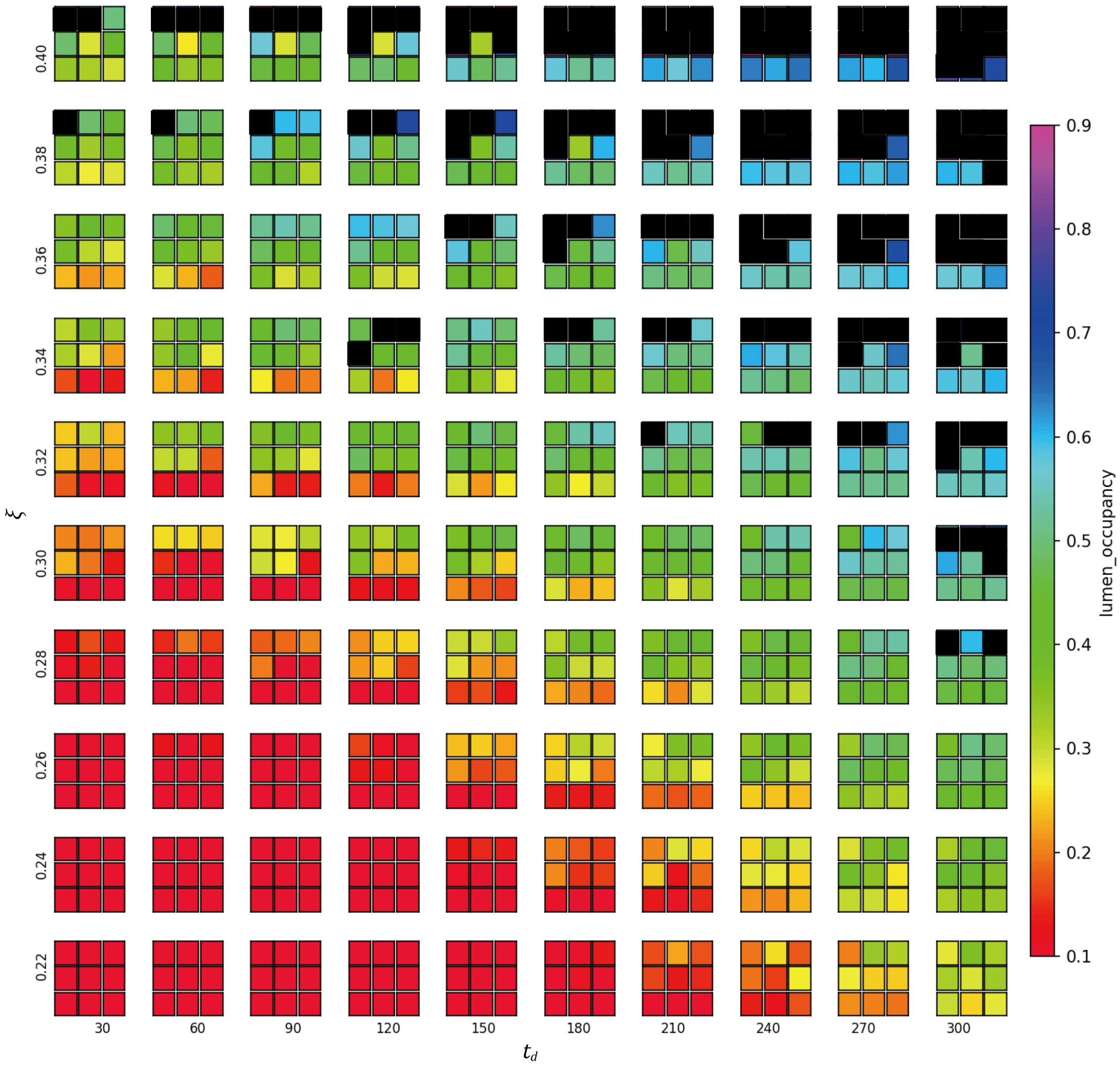

Fig M. Change in lumen occupancy for various parameter sets. Each mini-grid shows the change in lumen occupancy for nine η values at each ξ–t_d coordinate; color represents the change level. Black squares indicate the ruptured sheet state. The nine η values within each mini-grid are arranged as follows: the top row shows $\eta = 0.002$, 0.003, and 0.004 from left to right; the middle row shows 0.005, 0.006, and 0.007; and the bottom row shows 0.008, 0.009, and 0.010.

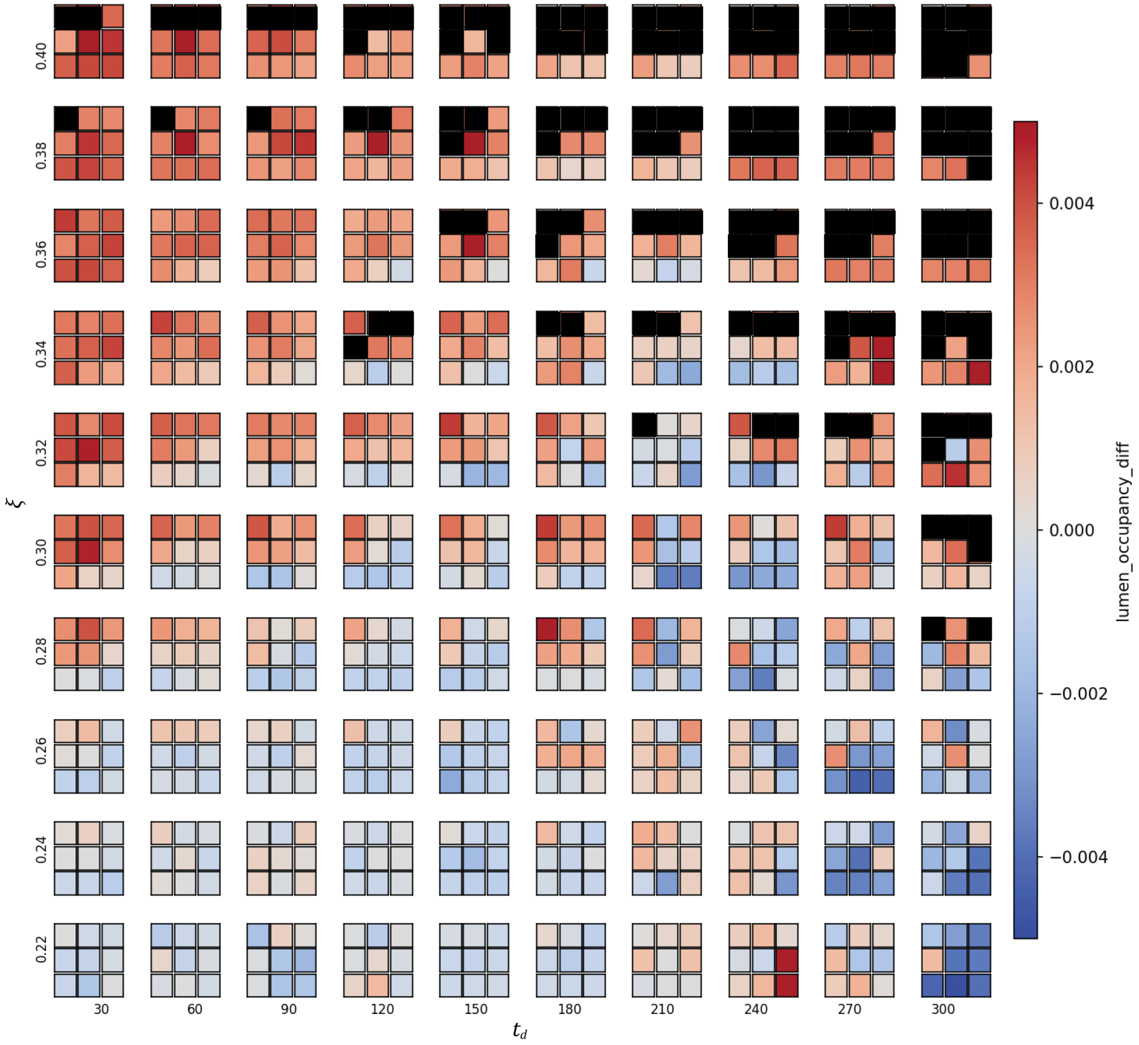

Fig N. Lumen index for various parameter sets. Each mini-grid shows the lumen index for nine η values at each ξ–t_d coordinate; color represents the index value. Black squares indicate the ruptured sheet state. The nine η values within each mini-grid are arranged as follows: the top row shows $\eta = 0.002$, 0.003, and 0.004 from left to right; the middle row shows 0.005, 0.006, and 0.007; and the bottom row shows 0.008, 0.009, and 0.010.

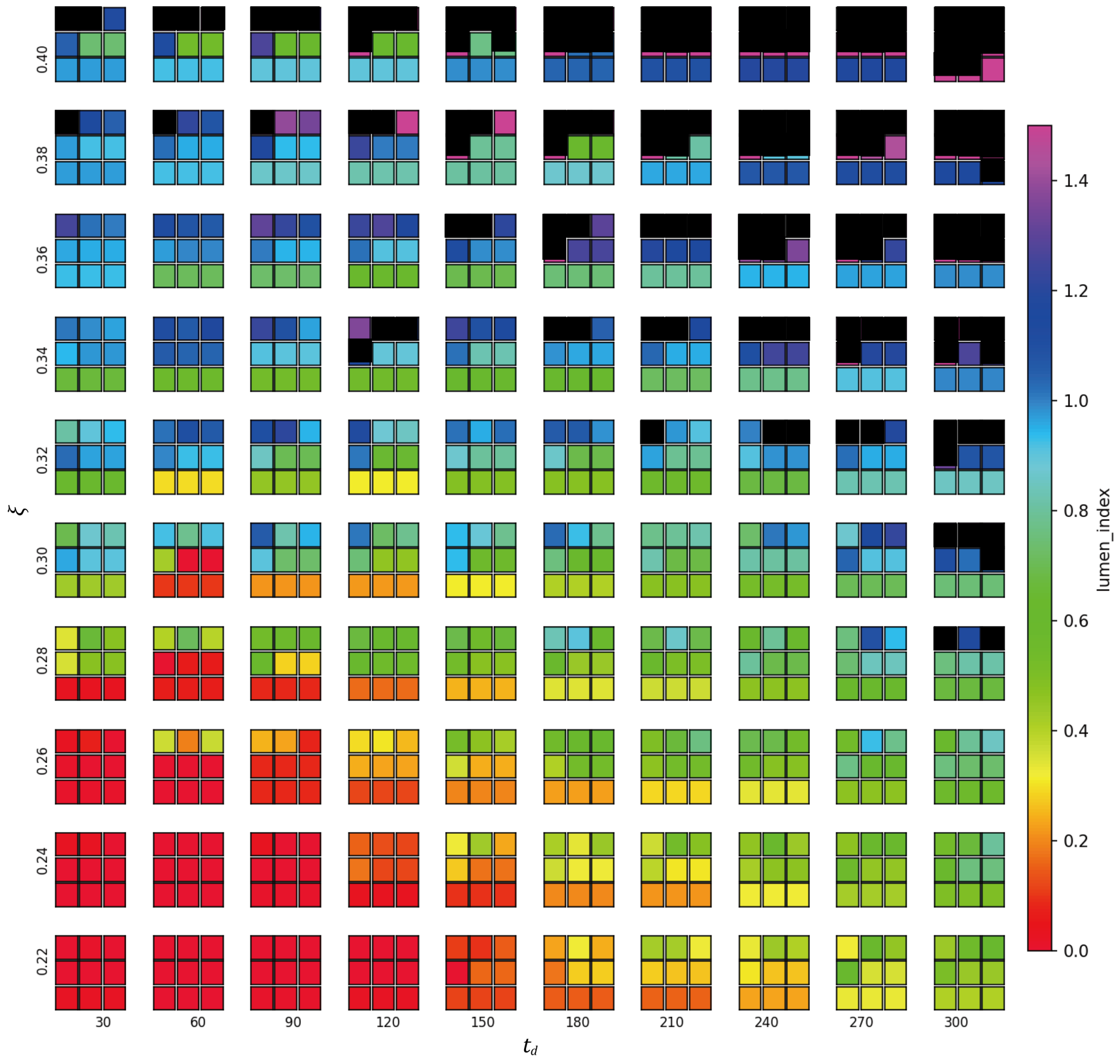

3 Initial cell numbers and morphology

Fig O. Morphologies resulting from different initial numbers of cells. The final states of the organoid when: (a) $(\xi, t_d) = (0.37, 0)$, (b) $(\xi, t_d) = (0.36, 280)$, (c) $(\xi, t_d) = (0.33, 100)$, (d) $(\xi, t_d) = (0.30, 120)$, (e) $(\xi, t_d) = (0.28, 60)$, and (f) $(\xi, t_d) = (0.28, 140)$. Each column corresponds to a different initial cell count.

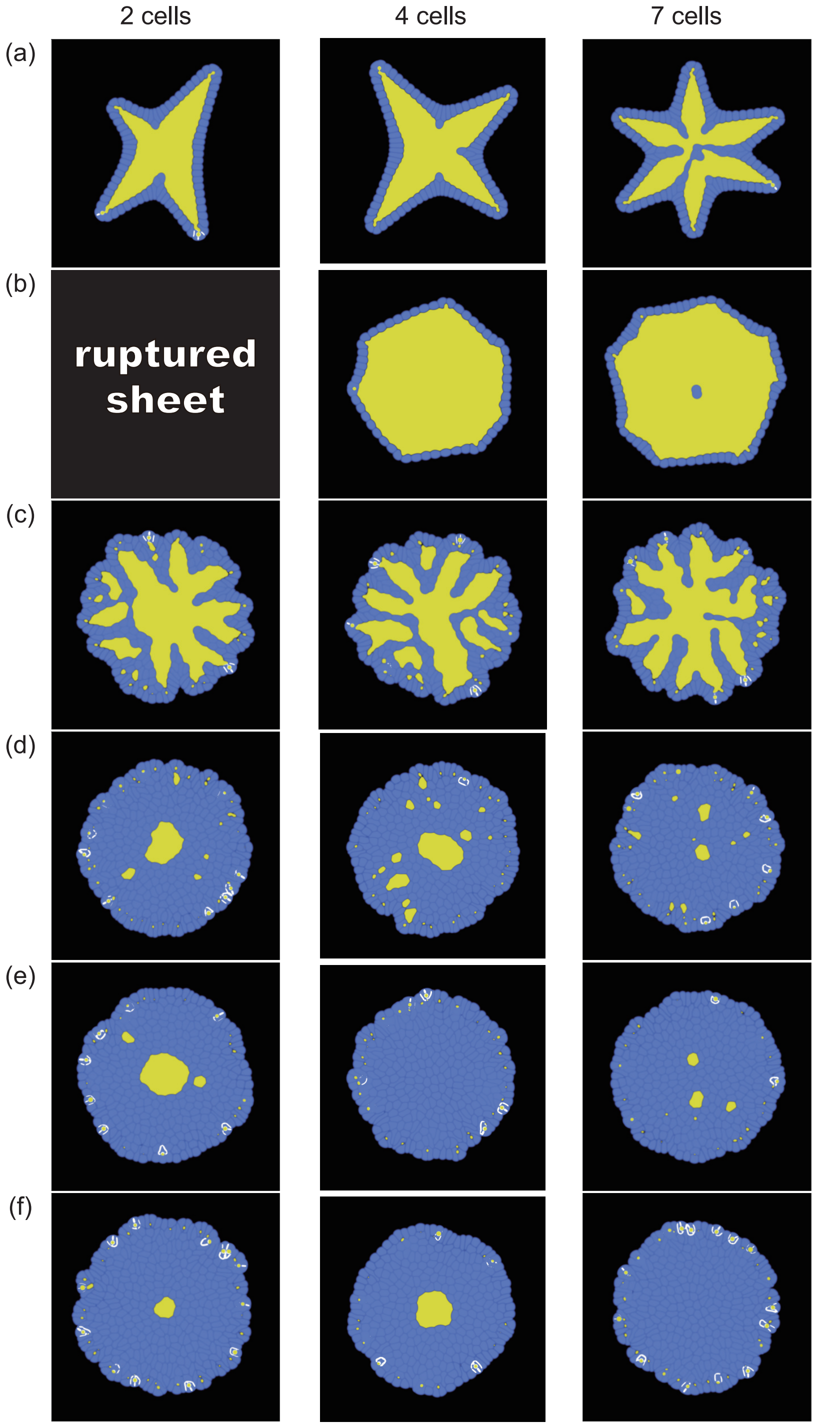

Beyond the parameters outlined in the main text, various factors can influence the morphology of organoids within our model. A critical factor is the number of initial cells. Given that there is no lumen at the outset, the morphologies of organoids from different initial cell counts can diverge as the cells proliferate.

Distinct morphological phases are observed depending on the initial number of cells, leading to varied organoid structures under specific conditions. Fig O showcases the morphologies of organoids that originated from different numbers of initial cells. The series in the center features organoids that started with four cells, consistent with the results shown in the main text. To its left and right are series starting with two and seven cells, respectively, with the latter arranged such that one cell is encircled by the other six.

A notable difference emerges in Fig O(b) by high lumen pressure and extended minimum cell cycle duration, where the organoid that began with two cells ruptures (left). This observation underscores the importance of a sufficient initial cell count against the lumen volume to maintain the monolayer integrity, particularly during the early stages.

Additionally, the number of branches in star-shaped organoids beginning with seven cells usually manifests as six branches, diverging from the four branches observed in other cases, as depicted in Fig O(a). The emergence of six branches in the organoid with seven initial cells is attributed to the initial configuration: six cells forming the outer layer with one cell positioned centrally. Organoids starting with two cells display four branches, deviating from the anticipated two, due to the insufficiency of two cells to enclose a single central lumen without deformation of cells. Our model, which excludes volume noise, suggests that the branched morphology is significantly influenced by the initial cellular arrangement.

Another variation is seen in Figs O(e) and O(f), under conditions of elevated lumen pressure. In Fig O(e) at $(\xi, t_d) = (0.28, 60)$, organoids with two and seven initial cells develop into a multilayered structure with multiple lumens (left and right), in contrast to a multilayered structure without stable lumens formed when starting with four cells (center). In Fig O(e) at $(\xi, t_d) = (0.28, 240)$, an organoid that began with seven cells displays a multilayered structure without stable lumens, unlike the multilayered structure with multiple lumens seen in others.
