# Supplementary figures and images for: Predicting organoid morphology through a phase field model: Insights into cell division and lumenal pressure
Source: PLoS Comput Biol. 2025 Aug 18;21(8):e1012090. doi: 10.1371/journal.pcbi.1012090 (PMC12373292; doi:10.1371/journal.pcbi.1012090)

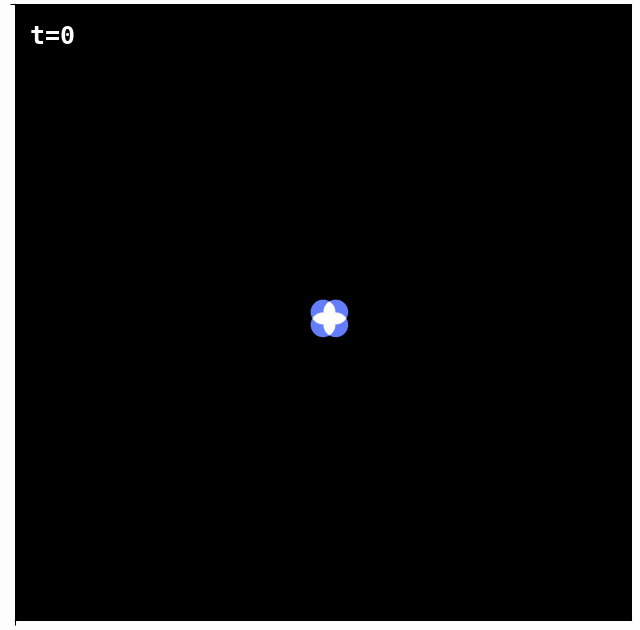

Supplement: S1 Movie — Simulation result showing the time evolution of a star-shaped organoid with parameters (ξ,td)=(0.37,0). (GIF) [file pcbi.1012090.s002.gif]

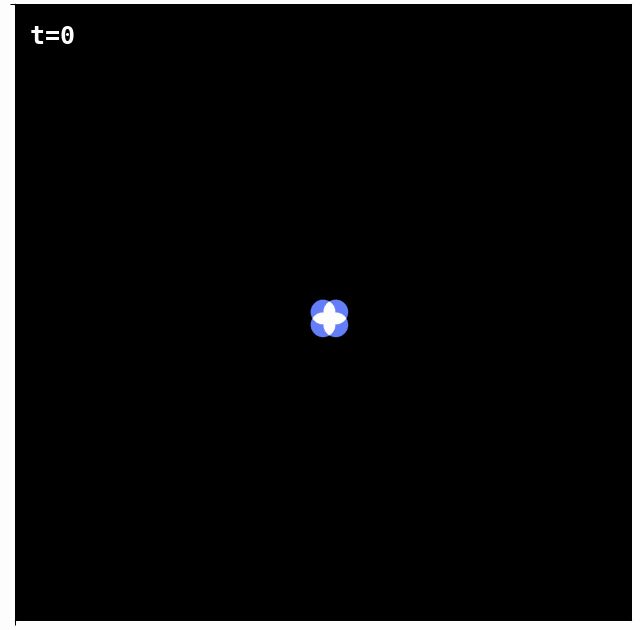

Supplement: S2 Movie — Simulation result showing the time evolution of a monolayer cyst organoid with parameters (ξ,td)=(0.36,280). (GIF) [file pcbi.1012090.s003.gif]

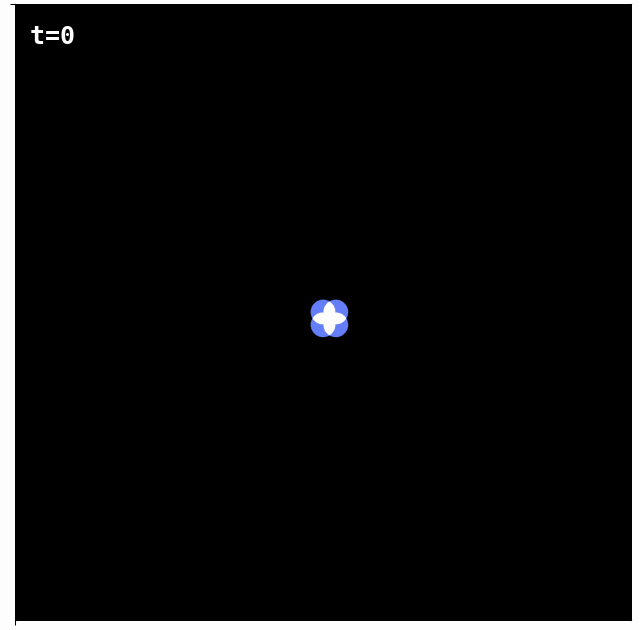

Supplement: S3 Movie — Simulation result showing the time evolution of a branched multi-lumen organoid with parameters (ξ,td)=(0.33,100). (GIF) [file pcbi.1012090.s004.gif]

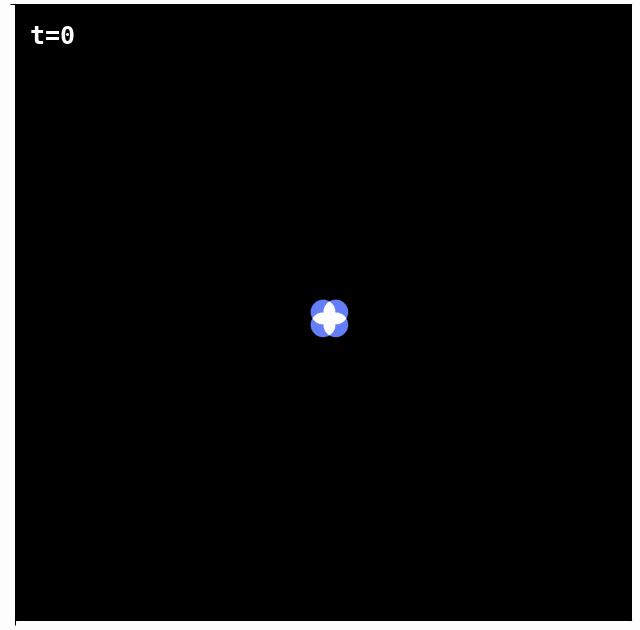

Supplement: S4 Movie — Simulation result showing the time evolution of a multilayer multi-lumen organoid with parameters (ξ,td)=(0.30,120). (GIF) [file pcbi.1012090.s005.gif]

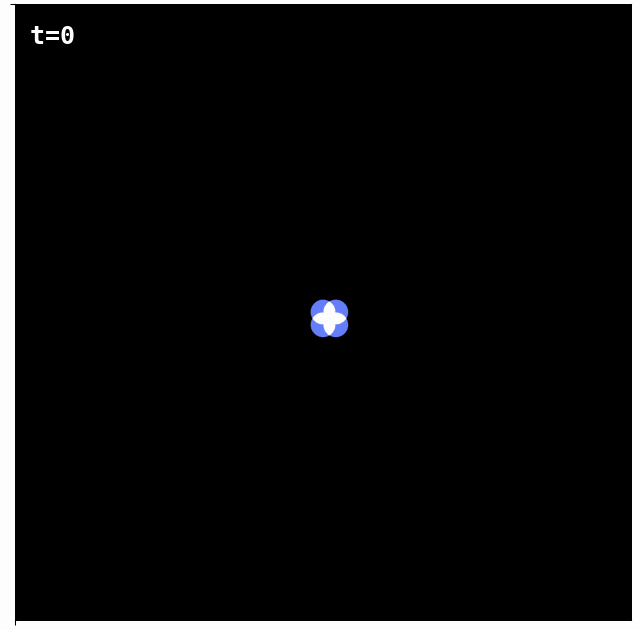

Supplement: S5 Movie — Simulation result showing the time evolution of a multilayer no-stable-lumen organoid with parameters (ξ,td)=(0.28,60). (GIF) [file pcbi.1012090.s006.gif]

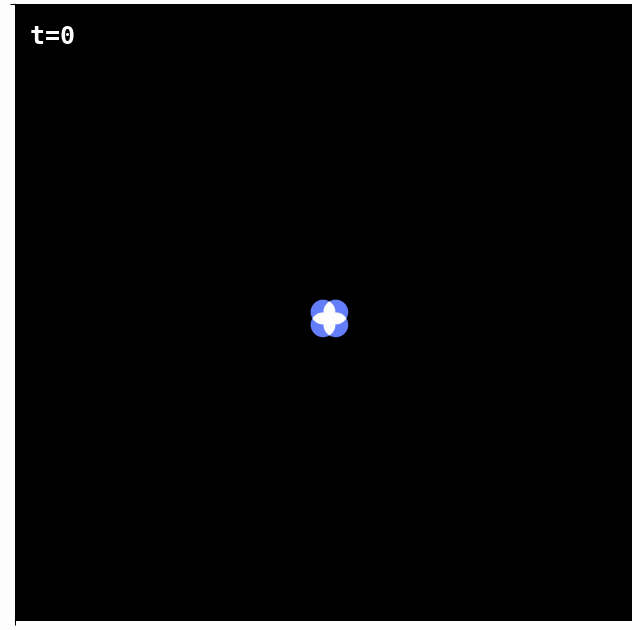

Supplement: S6 Movie — Simulation result showing the time evolution of a multilayer single-stable-lumen organoid with parameters (ξ,td)=(0.28,140). (GIF) [file pcbi.1012090.s007.gif]
